# Dereplication of Natural Products Using GC-TOF Mass Spectrometry: Improved Metabolite Identification by Spectral Deconvolution Ratio Analysis

**DOI:** 10.3389/fmolb.2016.00059

**Published:** 2016-09-30

**Authors:** Fausto Carnevale Neto, Alan C. Pilon, Denise M. Selegato, Rafael T. Freire, Haiwei Gu, Daniel Raftery, Norberto P. Lopes, Ian Castro-Gamboa

**Affiliations:** ^1^Núcleo de Pesquisas em Produtos Naturais e Sintéticos, Departamento de Física e Química, Faculdade de Ciências Farmacêuticas de Ribeirão Preto, Universidade de São PauloRibeirão Preto, Brazil; ^2^Núcleo de Bioensaios, Biossíntese e Ecofisiologia de Produtos Naturais, Departamento de Química Orgânica, Instituto de Química, Universidade Estadual Paulista UNESPAraraquara, Brazil; ^3^Centro de Imagens e Espectroscopia in vivo por Ressonância Magnética, Instituto de Física de São Carlos, Universidade de São PauloSão Carlos, Brazil; ^4^Department of Anesthesiology and Pain Medicine, Northwest Metabolomics Research Center, University of WashingtonSeattle, WA, USA; ^5^Jiangxi Key Laboratory for Mass Spectrometry and Instrumentation, East China Institute of TechnologyNanchang, China; ^6^Public Health Sciences Division, Fred Hutchinson Cancer Research CenterSeattle, WA, USA

**Keywords:** GC-MS, plant metabolomics, compound identification, peak deconvolution, ratio analysis

## Abstract

Dereplication based on hyphenated techniques has been extensively applied in plant metabolomics, thereby avoiding re-isolation of known natural products. However, due to the complex nature of biological samples and their large concentration range, dereplication requires the use of chemometric tools to comprehensively extract information from the acquired data. In this work we developed a reliable GC-MS-based method for the identification of non-targeted plant metabolites by combining the Ratio Analysis of Mass Spectrometry deconvolution tool (RAMSY) with Automated Mass Spectral Deconvolution and Identification System software (AMDIS). Plants species from Solanaceae, Chrysobalanaceae and Euphorbiaceae were selected as model systems due to their molecular diversity, ethnopharmacological potential, and economical value. The samples were analyzed by GC-MS after methoximation and silylation reactions. Dereplication was initiated with the use of a factorial design of experiments to determine the best AMDIS configuration for each sample, considering linear retention indices and mass spectral data. A heuristic factor (CDF, compound detection factor) was developed and applied to the AMDIS results in order to decrease the false-positive rates. Despite the enhancement in deconvolution and peak identification, the empirical AMDIS method was not able to fully deconvolute all GC-peaks, leading to low *MF* values and/or missing metabolites. RAMSY was applied as a complementary deconvolution method to AMDIS to peaks exhibiting substantial overlap, resulting in recovery of low-intensity co-eluted ions. The results from this combination of optimized AMDIS with RAMSY attested to the ability of this approach as an improved dereplication method for complex biological samples such as plant extracts.

## Introduction

Dereplication plays a crucial role in natural products discovery and plant metabolomics studies. It provides fast identification of known metabolites present in complex mixtures using small quantities of crude material and avoids time-consuming isolation procedures (Dinan, 2005).

Typically, dereplication studies are based on comparison of data originating from chromatographic and spectroscopic techniques, such as LC-UV, LC-MS, LC-MS/MS, and GC-MS, with molecular features present in standard compounds libraries such as Chapman and Hall's Dictionary of Natural Products, METLIN metabolite database, Pubchem, ChemSpider, Chemical Abstracts Services, or NAPRALERT. Dereplication utilizes orthogonal physicochemical characteristics, e.g., UV−Vis profiles, chromatographic retention times, molecular weight, NMR chemical shifts, or biological properties, in order to confirm the metabolic identification (Smith et al., 2005; Blunt and Munro, 2007; Lang et al., 2008).

Although this approach has proven to be very efficient for rapid identification of compounds in mixtures, it has some analytical limitations. Such limitations are mainly related to detection limits, lack of chromatographic resolution, or the need for additional confirmatory data such as MS/MS and NMR experiments (Motti et al., 2009; Tang et al., 2009; El-Elimat et al., 2013; van der Hooft et al., 2013).

In GC-MS analysis, the standard electron ionization (EI) at 70 eV provides reproducible and characteristic molecular ions and fragments. Molecular identification can be established by matching the spectral dataset with standard mass spectral databases, such as the National Institute of Standards and Technology (NIST), the Agilent Fiehn GC-MS Metabolomics Retention Time Locking (RTL) library, GOLM Metabolome Database (GMD), Wiley Mass Database, MoNA Database (http://mona.fiehnlab.ucdavis.edu) or others (Kopka et al., 2005; Babushok et al., 2007; Kind et al., 2009). Additionally, GC-retention time (Rt) reproducibility can be used as orthogonal information (to MS data) for compound identification (Kind et al., 2009).

Nevertheless, GC-MS-based metabolomics studies have important limitations when two or more molecules overlap chromatographically, especially because of the inherent hard fragmentation of EI (Du and Zeisel, 2013). Soft ionization techniques, such as chemical ionization (CI) can overcome this issue by preserving the molecular integrity and avoiding in-source fragmentation. Still, the lack of informative data (fragment ions) in CI hampers the rapid identification of known compounds (Andrade et al., 2008).

Recent advances in chemometric tools combined with the extensive compound libraries have made substantial progress in EI-based metabolic identification. In general, statistical analysis can extract essentially all relevant information from large datasets, even with high degrees of spectral overlap, allowing for the removal of noise and interferents (Pilon et al., 2013; Yang et al., 2013). AMDIS software has been employed in GC-MS data to deconvolute, recover and identify compounds based on peak shape and spectral information (Stein, 1999). Despite AMDIS's status as the most widely used deconvolution method for GC-MS, the indiscriminate use of its empirical parameters and arbitrary rules can generate as much as 70–80% false assignments (Lu et al., 2008; Likić, 2009).

More recently, an alternative statistical approach called Ratio Analysis of Mass Spectrometry (RAMSY) has been proposed, which facilitates compound identification via comparison between MS peak-intensities that form non-resolved chromatographic peaks. RAMSY can be utilized to analyze data from different platforms, including GC-MS and high resolution LC tandem MS, using data from distinct samples (Gu et al., 2013). In this study we developed a new GC-MS-based protocol for rapid identification of plant metabolites using the RAMSY deconvolution algorithm in combination with AMDIS deconvolution to provide an improved spectral identification workflow. The proposed method is initiated with the optimization of AMDIS deconvolution parameters using a fractional design of experiments, followed by the application of RAMSY as a “digital filter” for the AMDIS metabolite identification process. Solanaceae, Chrysobalanaceae, and Euphorbiaceae plant species were selected as model systems to evaluate the new metabolite identification process due to their ethnopharmacological potential and economical value (Sharma and Singh, 2012; Carnevale Neto et al., 2013; Funari et al., 2013; Zappi et al., 2015).

## Experimental methods

### Chemicals

A FAME mixture consisting of a set of 22 fatty acid methyl esters of chain lengths from C8–C30 was purchased in the form of the Fiehn GC/MS Metabolomics Standards Kit (7 ampoules: 1 × 0.5 mL FAME/*d*27 mixture, 1 × 0.5 mL pyridine, 1 × 0.5 mL MSTFA/1 % TMCS and 4 × 1.2 mL *d*27 mystric acids mix) from Agilent Technologies (Santa Clara, CA, USA). *O*-methylhydroxylamine hydrochloride, MSTFA (*N*-methyl-*N*-trifluoroacetamide) with 1% TMCS (trimethylchlorosilane), TSP (trimethylsilylpropionic acid-*d*4, sodium salt) and pyridine (silylation grade) were purchased from Sigma Aldrich (St Louis, MO, USA).

### Biological material

Plants samples were collected in several Ecological stations in São Paulo State, Brazil as part of the storage of 2,000 plant extracts by Brazilian Biodiversity Virtual Institute Program (www.biota.org.br). Giselda Durigan identified all species and vouchered specimens were deposited at the São Paulo State Botanical Institute herbarium (SP) as shown in Table 1.

**Table 1:** **Collection locations of plant species**.

**N°**	**Plant species**	**Part**	**Vourcher n°**	Brazilian ecological stations^*^
1	*Licania hoehnei*	Leaves	M847	Estação Ecológica da Juréia-Itatins/Núcleo Arpoador
2	*L. kunthiana*	Leaves	M846	Estação Ecológica da Juréia-Itatins/Núcleo Arpoador
3	*L. humilis*	Stems	Nu-Assis-87	Estação Ecológica e Experimental de Assis
4	*L. humilis*	Leaves	Nu-Assis-88	Estação Ecológica e Experimental de Assis
5	*Couepia grandiflora*	Leaves	Nu-Assis-85	Estação Ecológica e Experimental de Assis
6	*C. grandiflora*	Stems	Nu-Assis-86	Estação Ecológica e Experimental de Assis
7	*Hirtella hebeclada*	Leaves	M491	Parque Estadual da Serra do Mar/Núcleo Cunha
8	*H. hebeclada*	Leaves	M799	Estação Ecológica da Juréia-Itatins/Núcleo Arpoador
9	*H. hebeclada*	Stems	M851	Estação Ecológica da Juréia-Itatins/Núcleo Arpoador
10	*Parinari excelsa*	Leaves	M821	Estação Ecológica da Juréia-Itatins/Núcleo Arpoador
11	*Jatropha multifida*	Leaves	HRCB 43223	UNESP—Araraquara experimental garden
12	*J. gossypifolia*	Leaves	HRCB 43224	UNESP—Araraquara experimental garden
13	*Solanum swartzianum*	Leaves	R271	Estação Ecológica e Experimental de Assis
14	*S. swartzianum*	Stems	R272	Estação Ecológica e Experimental de Assis
15	*S. swartzianum*	Leaves	F052	Parque Estadual da Serra do Mar/Núcleo Cunha
16	*S. americanum*	Leaves	M951	Estação Ecológica de Itirapina
17	*S. americanum*	Stems	M952	Estação Ecológica de Itirapina
18	*S. excelsum*	Leaves	F55	Parque Estadual da Serra do Mar/Núcleo Cunha

1–10, Chrysobalanaceae; 11–12, Euphorbiaceae; 13–18; Solanaceae.

* The ecological stations where samples were collected are specifically protected areas of Brazil defined by the National System of Conservation Units (SNUC).

### Extraction procedure

The plants were separated into leaves and stems, dried at room temperature and ground in a Wiley mill. The extraction procedure was chosen on the basis of similar conditions previously reported (Ju and Howard, 2003; Bergeron et al., 2005). Extractions were conducted using a Dionex ASE 100 system (Oakville, ON, Canada) with stainless steel vessels (66 mL) using 0.5 g of dry ground plant, and ~60 mL EtOH at 60°C and 1500 psi for 15 min. The extracts were dried using a vacuum evaporator (Eppendorf, Hauppauge, NY, USA).

### Sample preparation

All samples underwent a two-step derivatization procedure before GC-MS analysis (Gu et al., 2013). Initially, methoximation was performed to protect aldehydes and ketones and to inhibit the ring formation of reducing sugars. *O*-methylhydroxylamine hydrochloride solution (10 μL), prepared using 40 mg mL^−1^ *O*-methylhydroxylamine hydrochloride, (Sigma-Aldrich no. 226904—98.0%) in pyridine (99.9%) was added to the samples, and the mixtures were kept at 30°C for 90 min. Next, 90 μL *N*-methyl-*N*-trimethylsilyltrifluoroacetamine with 1% chlorotrimethylsilane (MSTFA+1% TMCS) was added to the samples and kept at 37°C for 30 min to allow the trimethylsilylation of acidic protons. Subsequently, 2.0 μL of the FAME mixture was added to each sample to provide retention time indices. The solutions were vortexed and transferred to GC-MS glass vials for analysis (Kind et al., 2009; Gu et al., 2013).

### GC-MS

Experiments were performed on an Agilent 7890A GC-5975C MSD system (Agilent Technologies, Santa Clara, CA) using a DB5-MS+10m Duraguard Capillary Column (30 m × 250 μm × 0.25 μm) as the stationary phase. The GC parameters used were as follows: split injection (1.0 μL sample at 100.0°C, 1.0 min—split ratio of 10:1); He carrier gas (40 cm s^−1^ at constant velocity); 275.0°C transfer line temperature; oven temperature program: 1.0 min at 100°C, increased 20.0°C min^−1^ to 200.0°C, then increased 3.0°C min^−1^ to 325.0°C and held for 10.0 min, MS parameters: electron impact ionization at 70 eV, filament source temperature of 230.0°C, quadrupole temperature of 150.0°C, m/z scan range 50–600 at 2 spectra s^−1^. Mass spectral signals were recorded after a 6.10 min solvent delay to avoid derivatization interferents, and turned off between 10.0 and 13.0 min to avoid saturation of the detector due to the high content of monosaccharides. A blank sample with the FAME standard mixture (FAME std) was also injected under the same GC conditions.

### AMDIS

The analysis of the full-scan data files acquired by GC-MS was performed using the empirical method developed by Dromey et al. (1976) and employed by Stein (1999) using the AMDIS software package. The overall deconvolution process in AMDIS consists of three sequential steps: (1) noise analysis, (2) component perception and (3) model shape determination and spectrum deconvolution (Stein, 1999). The first step extracts the noise from GC-MS data file by empirically calculating a noise factor using the median abundance level of a representative background segment obtained from 13 scans.

Component perception (2), identifies individual chromatographic components by considering ions that have maximal intensities at the same time (i.e., the same or similar scan). It is achieved through a process which sequentially examines individual peak maxima using a pre-set number of scans (4–32) in the forward and reverse directions.

Step (3) determines the peak shape for each perceived component and performs the extraction of “pure” MS spectra. A peak shape model is determined by considering the sum of individual ion chromatograms that maximize together and that have sharpness values within 75% of the maximum value for a particular component, as defined by:

(1) $$\begin{array}{l} sharpness=\frac{\left(A_{\max}-A_{n}\right)}{\left(n\times N_{f}\sqrt{A_{\max}}\right)} \end{array}$$

where *A*_max_ is the maximum abundance, *A*_*n*_ is the abundance from a pre-set number of scans *n* and *N*_*f*_ is noise factor. Finally, the spectrum is recovered using a least-squares method where each *m/z* value is individually fit to the model profile, allowing a linear baseline:

(2) $$\begin{array}{l} A_{\left(n\right)}=a+b\times n+c\times M_{\left(n\right)}+d\times Y_{\left(n\right)} \end{array}$$

where *A*_(*n*)_ is the abundance during scan *n*. *a, b*, and *c* are derived constants, and *M*_(*n*)_ is the abundance of the model profile during scan *n.* Box 1 illustrates the metabolic identification process using AMDIS.

Box 1 **Amdis approach.**

**Figure:**
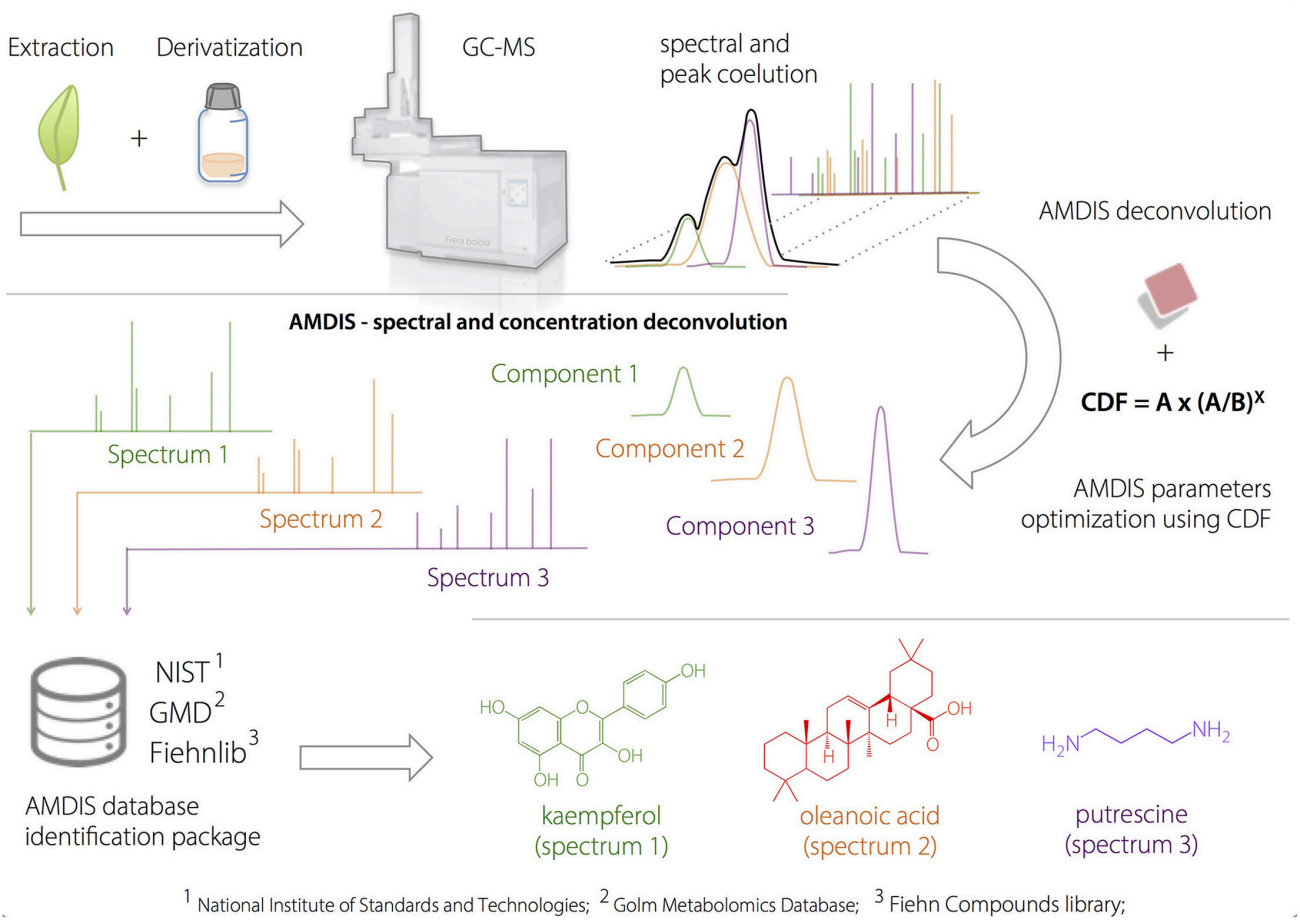
**Application of AMDIS Software to the Deconvolution of Metabolites in Plant Samples**.

Plant extracts were injected into the GC-MS after a two-step derivatization process. AMDIS deconvolution was optimized according to a developed heuristic correction factor (compound detection factor, CDF), to prevent false-positive identification. After the GC-deconvolution using AMDIS-based optimized parameters, the putative identification was performed by spectral comparison (based on match factor, *MF*) with available compound databases and using linear retention indices as orthogonal information.

### RAMSY

Ratio analysis is designed to work using single datasets that contain multiple MS spectra for the same metabolite (Gu et al., 2013). For peaks that originate from the same compound, under the same experimental conditions, their MS peak intensity ratios across the chromatographic peak should be relatively constant. In addition, the standard deviations of those ratios should be small (zero in principle; Gu et al., 2013).

The procedure for calculating the RAMSY spectrum was described previously (Gu et al., 2013). Briefly, one isolated peak in the mass spectrum is selected as the driving peak. The intensity value of this selected driving peak is divided by the intensities of all the other peaks in the spectra one at a time, as shown in Equation (3):

(3) $$\begin{array}{l} D_{i,j}=\frac{X_{i,j}}{X_{i,k}} \end{array}$$

where, the vector *X*_*i*_ is the *i*th spectrum of a set of *n* MS spectra, and the *j*th data point of *m* total points in that spectrum is denoted as *X*_*i, j*_ (*X*_*i, k*_ is the driving peak). *D* is the ratio matrix of dimension *n* × *m*.

The RAMSY values, denoted as an *m*-element vector *R*, are the quotients of means and standard deviations across columns of *D*. The standard deviation is zero for the driving peak itself; therefore, its RAMSY value is pre-defined (e.g., the value of the highest RAMSY ratio). The other RAMSY values are calculated as elements of the vector *R* as follows:

(4) $$\begin{array}{l} {\text{R}}_{j}=\frac{\frac{1}{n}\sum_{i=1}^{n}{\text{D}}_{i,j}}{\sqrt{\frac{1}{n}\sum_{i=1}^{n}{\left({\text{D}}_{i,j}-\frac{1}{n}\sum_{i=1}^{n}{\text{D}}_{i,j}\right)}^{2}}} \end{array}$$

Since a ratio's standard deviation is used as the denominator, a small standard deviation will produce a large reciprocal value, generating a peak (in principle an MS peak from the same compound as that for the driving peak). In general, the MS peaks from interfering compounds will generate large standard deviations and thus small RAMSY numbers, similar to noise values. Notably, RAMSY values are dimensionless.

### Data analysis

**Figure 1:**
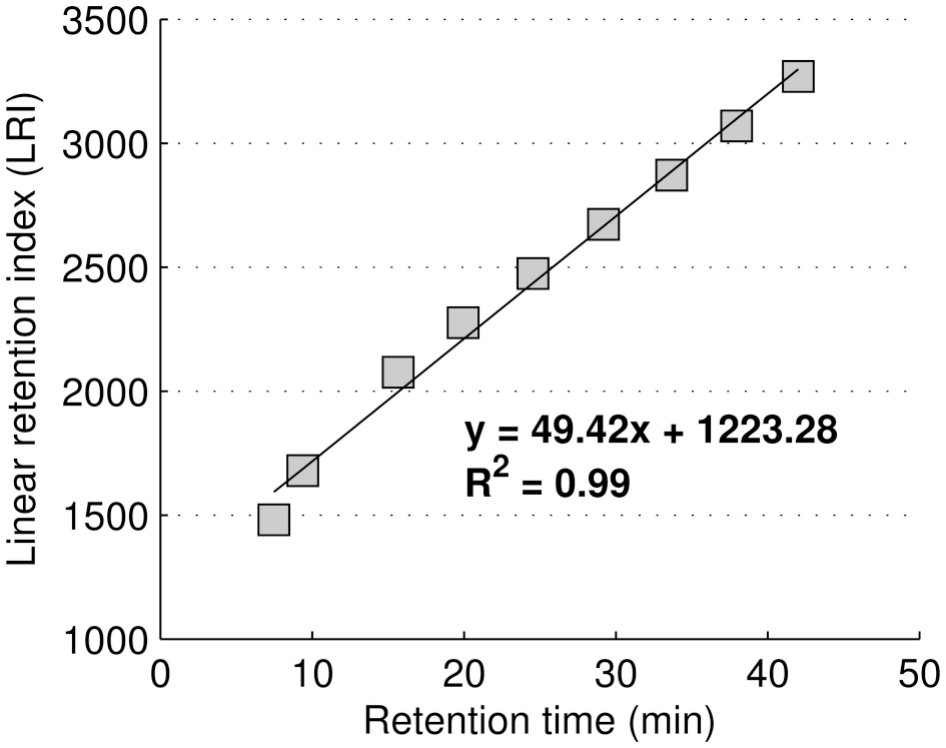
**Regression equation used to calculate the linear retention index using FAME internal standards**.

GC-MS metabolite identification was performed considering two independent parameters: the Linear Retention Index (LRI) and the mass spectrum (MS) similarity profile. LRIs were based on linear regression using FAME internal standards retention times according to Van den Dool and Kratz, and are shown in Figure 1 (Van Den Dool and Kratz, 1963).

The MS similarity profiles were calculated by comparison of AMDIS and RAMSY deconvoluted spectra using two MS databases, FiehnLib (University of California, Davis, CA, USA, Agilent webpage) and the Golm Metabolome database (Max Planck Institute, Potsdam–Golm, Germany). The similarities between samples and databases were calculated using the same algorithm as those reported for the NIST library (Stein and Scott, 1994). Briefly, we first obtained the “angle” between the two spectra:

(5) $$\begin{array}{l} F_{1}=\frac{{\sum M\left(A_{S}A_{U}\right)}^{1/2}}{{\left[\sum MA_{S}\sum MA_{U}\right]}^{1/2}} \end{array}$$

*M* is the *m/z* value, and *A*_*S*_ and *A*_*U*_ are the base-peak normalized abundances of the peaks in the standard spectrum and unknown spectrum, respectively. Next, *F*_2_ is calculated:

(6) $$\begin{array}{l} F_{2}=\left(\frac{1}{N_{U\&S}-1}\right)\overset{N_{U\&S}}{\underset{i=2}{\sum }}{\left(\frac{A_{S,i}}{A_{S,i-1}}\right)}^{n}{\left(\frac{A_{U,i}}{A_{U,i-1}}\right)}^{-n} \end{array}$$

*F*_2_ is based on relative intensities of pairs of adjacent peaks present in both spectra. *N*_*U&S*_ is the number of peaks common to the unknown and standard spectra, and *n* = 1 (−1) if the first abundance ratio is less (larger) than the second. The Match Factor (*MF*) is then calculated as follows:

(7) $$\begin{array}{l} MF=\frac{1000}{N_{U}+N_{U\&S}}\left(N_{U}F_{1}+N_{U\&S}F_{2}\right) \end{array}$$

A perfect match results in an *MF*-value of 1000; spectra with no peaks in common result in a value of 0.

Previous studies on automated metabolite identification efficacy using AMDIS showed relatively high (27.8–32.8% false positive rates (on average) using *MF* 700–900 (Aggio et al., 2014). For that reason, we considered a positive identification only with LRI errors ≤ 5% and MS similarity profiles with *MF* ≥ 700. The RAMSY algorithm was applied for chromatographic regions with detected metabolite *MF*s in the range of 700–790. Box 2 illustrates the metabolic identification process using AMDIS-RAMSY.

Box 2 **Ramsy approach.**

**Figure:**
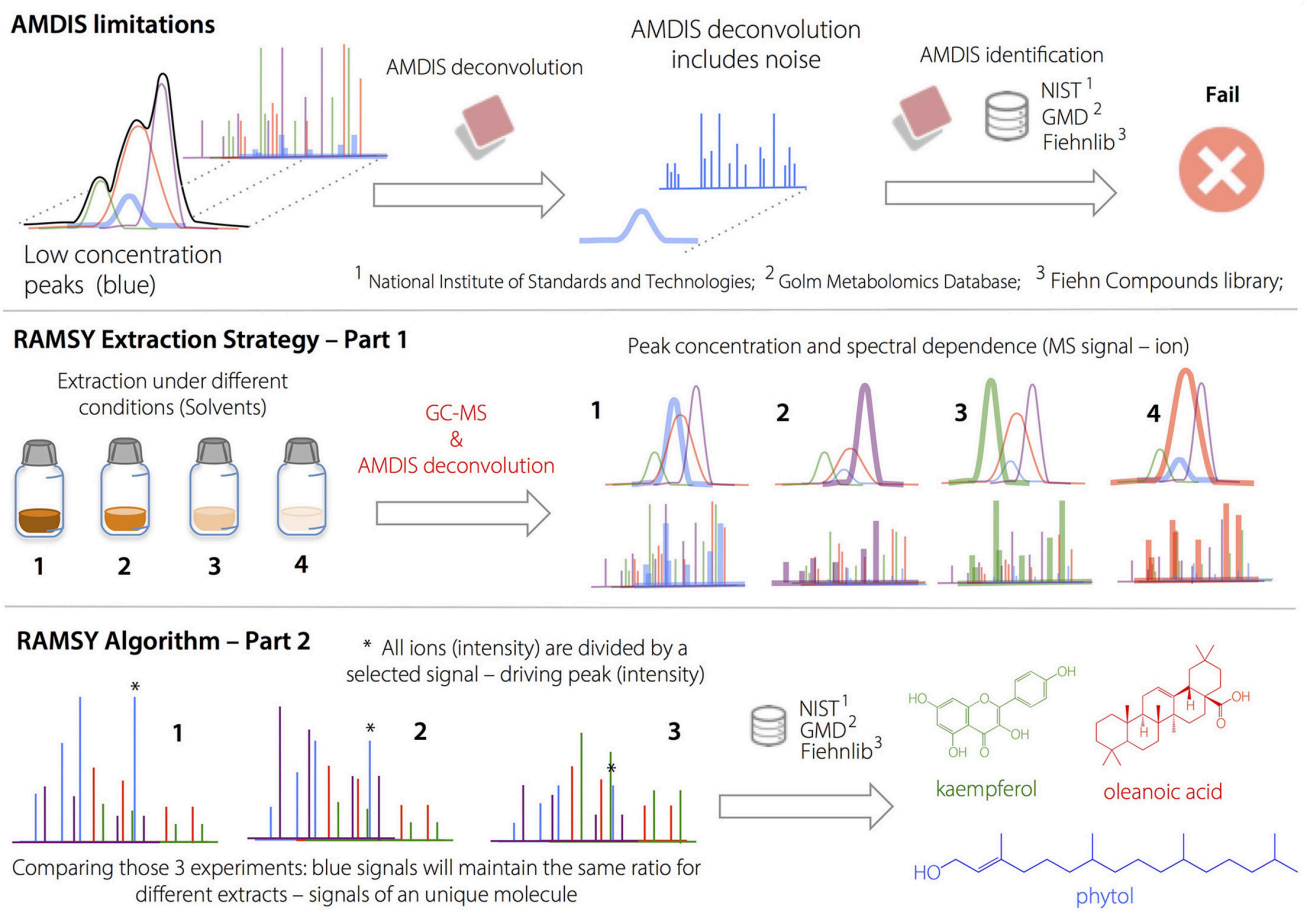
**Application of RAMSY to the Deconvolution of Overlapped Peaks after AMDIS**.

AMDIS limitations on deconvolution led to low MF values and/or missing metabolites in regions with high peak overlap. The deconvolution and identification of major metabolites and well-resolved GC peaks by AMDIS was followed by the application of the RAMSY algorithm described in the text. In RAMSY, the quotients of average peak ratios and their standard deviations using all the MS scans from the same ion chromatogram efficiently allow the statistical recovery of the metabolite peaks and facilitate reliable identification. RAMSY was applied to peaks exhibiting substantial overlap, resulting in the recovery of low-intensity and co-eluted ions as well as an improvement to the AMDIS deconvolution process.

### Chemometric analysis

#### 2*v*^5−1^ fractional design

In order to evaluate the AMDIS deconvolution parameters, a two-level fractional factorial design (2*v*^5−1^) was applied (Brereton, 2007). The component width (8 or 32), number of adjacent peaks (0 or 2), resolution (low or high), sensitivity (low or high) and shape requirements (low or high) were evaluated according to a compounds detection factor (CDF). The effects (variables) were fitted at the 95% confidence level. The meaningful variables for each sample are show in Table 2. All data were calculated using Excel 365 Home (Microsoft Office, USA).

**Table 2:** **Optimized AMDIS deconvolution and identification parameters using CDF**.

**Family**	**Species**	**AMDIS deconvolution parameters**					**Meaningful parameters**
		**V1**	**V2**	**V3**	**V4**	**V5**	
Chrysobalanaceae	*Couepia grandiflora*^L^	−1	1	−1	−1	−1	V4, V5, V2-4, V2
Chrysobalanaceae	*C. grandiflora*^S^	−1	1	−1	−1	−1	V4, V2
Chrysobalanaceae	*Hirtella hebeclada*^L^	−1	1	−1	−1	−1	V4, V4-5, V2
Chrysobalanaceae	*H. hebeclada*^L^	−1	1	1	−1	1	V4, V2-4, V2
Chrysobalanaceae	*H. hebeclada*^S^	−1	1	−1	−1	1	V4, V3, V2
Chrysobalanaceae	*Licania hoehnei*^L^	−1	1	1	−1	1	V4, V2
Chrysobalanaceae	*L. humilis*^B^	−1	1	−1	−1	−1	V4, V2
Chrysobalanaceae	*L. humilis*^L^	−1	1	−1	−1	−1	V4, V3, V5, V2, V4-5
Chrysobalanaceae	*L. kunthiana*^L^	−1	1	−1	−1	1	V4, V2-4, V2
Chrysobalanaceae	*Parinari excelsa*^L^	−1	1	−1	−1	−1	V4, V5, V2, V4-5
Euphorbiaceae	*J. gossypiifolia*^L^	−1	1	−1	−1	−1	V4, V5, V2-4, V2
Euphorbiaceae	*J. multifida*^L^	−1	1	−1	−1	1	V4, V3, V2
Solanaceae	*S. americanum*^L^	−1	1	1	−1	−1	V2, V3, V4, V5
Solanaceae	*Solanum americanum*^S^	−1	1	1	−1	−1	V2, V3, V4, V5
Solanaceae	*S. excelsum*^L^	−1	1	1	−1	−1	V2, V3, V4, V5
Solanaceae	*S. swartzianum*^S^	−1	1	1	−1	1	V4, V2-4, V3-4, V2, V3
Solanaceae	*S. swartzianum*^L^^*^	−1	1	1	−1	−1	V4, V2-4, V2
Solanaceae	*S. swartzianum*^L^^**^	−1	1	1	−1	−1	V4, V2

* Harvested in Cardoso city—Sao Paulo, Brazil.

** Harvested in Cunha city—São Paulo, Brazil.

L, leaves; S, stem; B, bark; V1, component with (−1 represent 8 scans and +1 represent 32 scans per section); V2, adjacent peak subtraction (−1 represent 0 and +1 represent 2 peaks per section); V3, resolution (−1 represent low and +1 represent high); V4, sensitivity (−1 represent low and +1 represent high); V5, shape requirements (−1 represent low and +1 represent high).

#### Hierarchical cluster analysis—(HCA)

The GC-MS raw data and the processed metabolite profiles using the default and optimized AMDIS parameters were subjected to hierarchical cluster analysis (HCA). The data matrices were autoscaled to the total area for each chromatogram and the HCA distance was measured according to the Canberra metric, using R software (v 3.03, R: A Language and Environment for Statistical Computing, Vienna, Austria, http://www.r-project.org/). In case of GC-MS raw data, the area of each peak, after being recognized and aligned, was autoscaled to the total area for each chromatogram using the XCMS R-package (Smith et al., 2006).

## Results and discussion

### Metabolic identification using AMDIS

The use of computational methods can assist the identification of known metabolites by extracting the signals from co-eluted GC-MS components (Du and Zeisel, 2013). AMDIS is a freely available software package that applies arbitrary rules for peak deconvolution and performs identification in an integrated matching system combined with the NIST standard reference and others databases (Stein, 1999). The designed dereplication protocol started with the application of a 2*v*^5−1^ fractional design on AMDIS parameters: component width (8 or 32), number of adjacent peaks (0 or 2), resolution (low or high), sensitivity (low or high) and shape requirements (low or high). The results were evaluated according to the compound detection factor (CDF) calculated by the proposed heuristic Equation (8),

(8) $$\begin{array}{r} CDF=A\times {\left(\frac{A}{B}\right)}^{x} \end{array}$$

CDF provides the optimized ratio between the number of detected (*A*) and identified compounds (*B*) by reducing the negative effects of variable over-fitting due to the inclusion of noise and/or false components. “*A*” represents the identification power derived from the library dataset extension, while the “(*A*/*B*)^*x*^” ratio expresses a penalty to conditions where a large number of peaks are detected but not identified. The “*x*” value depends on how important the constraint factor is to the model (typically *x* = 3). The best results occur when the relation between “*A*” and “*B*” is simultaneously increased. On the other hand, when only “*B*” is increased, CDF is reduced. The optimized deconvolution and identification parameters by AMDIS using the CDF are shown in Table 2.

The CDF indicated that high “adjacent peak subtraction,” low “component width,” and low “sensitivity” generated the best deconvoluted chromatograms, regardless of the sample, whereas “resolution” and “shape requirements” showed particular response based on the metabolic composition.

Optimized AMDIS deconvolution yielded ~200 components per sample, of which ~100 were putatively identified based on mass spectral *MF* and LRI correlations, as shown in Table 3. In general, the components that could be identified included amino acids, organic acids, fatty acids, carbohydrates, sugar alcohols, phytosterols, and specialized metabolites such as flavonoids, triterpenes, phenolics, polyamines, and related compounds.

**Table 3:** **Metabolites detected by GC-MS using AMDIS-RAMSY deconvolution**.

**Metabolite**	**Rt (min)**	**KI_lit_**	**KI_cal_**	**error (%)**	**1**	**2**	**3**	**4**	**5**	**6**	**7**	**8**	**9**	**10**	**11**	**12**	**13**	**14**	**15**	**16**	**17**	**18**
**PROTEIC AMINO ACIDS**																						
Serine	6.2	1354	1355	0.1	−	−	−	−	−	−	−	−	−	−	−	−	−	+	+	+	+	−
*L*-threonine	6.4	1377	1374	−0.2	−	−	−	−	−	−	−	−	−	−	−	−	−	−	−	+	+	−
Alanine	6.7	1424	1413	−0.8	−	−	−	−	−	−	−	−	−	−	+	−	−	+	+	+	+	+
*L*-aspartic acid	7.4	1511	1479	−2.2	−	−	−	−	−	−	−	−	−	−	−	−	−	+	−	−	−	−
Glutamic acid	8.2	1629	1615	−0.1	−	−	−	−	−	−	−	−	−	+	−	−	−	−	−	−	−	−
*L*-asparagine	8.7	1666	1606	−3.6	−	−	−	−	−	−	−	−	−	−	−	−	−	−	−	+	−	−
**NON-PROTEIC AMINO ACIDS**																						
Pipecolic acid	6.4	1365	1371	0.4	−	−	−	−	−	−	−	−	−	−	+	−	−	+	+	−	−	−
Pyroglutamic acid	7.5	1593	1522	−0.7	+	+	+	+	+	+	+	+	+	+	−	−	+	+	+	+	+	+
4-aminobutyric acid	7.5	1527	1493	−2.2	−	−	−	−	−	−	−	−	−	−	+	+	−	+	−	−	−	−
4-guanidinobutyric acid	7.5	1528	1494	−2.2	−	−	−	−	−	−	−	−	−	−	−	+	−	−	−	+	−	+
Butanoic acid, 4-amino	7.5	1594	1527	−0.7	−	+	+	+	+	+	+	−	+	+	−	−	−	−	−	−	−	−
**ORGANIC ACIDS**																						
Malonic acid	6.6	1479	1460	−1.1	−	−	−	−	−	−	−	−	−	−	−	+	−	−	−	+	−	−
Malic acid	7.1	1574	1479	−1	+	+	+	+	+	+	+	+	+	+	+	+	+	+	+	+	+	+
Trihydroxybutyric acid	7.5	^*^	1495	−	−	−	−	−	−	−	−	−	−	−	−	−	−	+	+	−	−	+
*L*-phenyllatic acid	7.6	1585	1539	−2.9	−	−	−	−	−	−	−	−	−	−	−	−	−	−	+	−	−	−
Threonic acid	7.7	1602	1545	−0.6	+	−	+	−	−	+	+	+	+	−	+	−	+	+	−	−	−	+
Cinnamic acid, trans	7.8	1607	1557	−0.5	−	−	−	−	−	−	−	−	−	+	−	−	−	−	−	−	−	−
Tartaric acid	8.3	1635	1629	−0.1	−	−	−	−	−	−	−	−	+	−	−	−	−	−	−	−	−	−
**PHENOLICS**																						
4-hydroxy-benzoic acid	8.4	1637	1633	0	+	−	+	−	−	+	−	+	+	+	−	−	+	+	+	+	+	+
4-hydroxyfenylacetic acid	8.5	1644	1596	−2.9	−	−	−	−	−	−	−	−	−	−	−	−	−	−	−	−	−	−
3-(2-hydroxyphenyl) propanoic acid	8.9	1670	1651	−0.8	−	−	−	−	−	−	−	−	−	−	−	−	−	−	−	+	−	−
Vanillic acid	9.8	1707	1766	0.6	−	−	+	+	+	+	−	−	−	−	+	+	+	−	−	+	−	+
4-hydroxy-3-methoxybenzoic	9.8	1707	1765	0.6	−	−	−	−	−	−	−	−	−	−	−	−	+	+	+	+	+	+
Ferulic acid	15	1962	1919	−0.4	+	−	−	−	−	−	−	−	−	−	−	+	−	−	−	−	−	+
Caffeic acid	15.8	2135	2114	−0.3	−	−	−	−	−	−	−	−	−	−	−	−	+	−	−	+	−	−
Chlorogenic acid	37.9	3099	3078	−0.2	−	−	−	−	−	−	−	−	−	−	−	−	−	+	+	+	−	−
Hydroquinone	6.6	1548	1402	−1.5	−	−	−	−	−	−	−	−	−	+	+	−	−	−	−	−	−	−
**POLYOLS**																						
*D*-threitol	7.3	1581	1485	−1	+	+	+	+	+	+	+	+	+	+	−	−	+	+	+	+	+	+
Erythritol	7.3	1581	1493	−0.9	+	+	+	+	+	+	+	+	+	+	−	−	+	+	+	+	+	+
Glycerol 1-phosphate	9.8	1714	1566	0.5	−	−	−	−	−	−	−	−	−	−	−	−	−	+	+	+	+	+
Ononitol	13.2	1875	1946	0.7	−	−	−	+	+	−	−	+	−	−	+	−	−	−	−	−	−	−
Myo-inositol	14.9	1957	2080	1.2	+	+	+	−	−	+	+	+	+	+	−	+	+	+	+	+	+	+
**CARBOHYDRATES**																						
Ribose	8.6	1646	1650	0	+	+	+	+	+	+	+	+	+	+	+	+	+	+	−	−	−	−
Arabinose	8.6	1646	1651	0	+	+	+	+	+	+	+	+	+	+	−	−	−	−	−	−	−	−
Xylose	8.6	1646	1646	0	+	+	+	+	−	+	+	+	+	+	−	−	−	−	−	−	−	−
Ribitol	9.2	1677	1713	0.4	−	+	+	+	+	+	+	+	+	+	+	+	+	+	+	+	+	+
Xylitol	9.2	1677	1695	0.2	+	+	+	+	+	+	+	+	−	+	+	+	+	+	−	−	+	−
Arabitol	9.2	1677	1708	0.3	+	+	+	+	+	+	+	+	+	+	−	+	+	+	+	+	+	+
Rhamnose	9.3	1682	1707	0.3	−	−	−	−	−	−	−	−	−	−	+	−	−	−	−	−	−	−
α,α-trehalose	29.3	2671	2726	0.6	−	+	+	+	−	−	+	+	+	−	+	+	+	+	+	+	+	+
Maltose	30	2705	2720	0.2	+	+	+	+	−	+	+	+	−	+	+	−	−	−	−	−	−	−
Melibiose	32.1	2809	2837	0.3	+	−	+	−	−	+	−	−	−	−	+	−	+	−	+	−	+	−
Isomaltose	32.2	2816	2847	0.3	+	−	+	+	−	+	+	−	−	+	−	−	−	−	−	−	−	−
Raffinose	37.9	3094	3075	−0.2	−	+	−	−	−	+	+	+	−	+	+	−	−	−	−	−	−	−
**POLYAMINES**																						
*L*-putrescine	9.5	1695	1737	0.4	−	−	−	−	−	−	−	−	+	−	+	−	−	−	−	+	−	−
**PURINES**																						
Xanthine	13.5	1890	2017	1.3	−	−	−	−	+	−	−	−	−	−	−	−	−	−	−	−	−	−
Uric acid	14.9	1961	2095	1.3	−	−	−	−	+	−	−	+	−	−	−	−	−	−	−	−	−	−
**FATTY ACIDS**																						
Hexadecanoic acid	14.1	1919	2046	1.3	+	+	+	+	+	+	+	−	+	−	−	−	+	+	+	+	+	+
Palmitic acid	14.1	1919	2048	1.3	−	−	−	−	−	−	−	−	−	−	−	−	−	+	+	−	−	−
Linoleic acid	17.4	2219	2166	−2.4	−	−	−	−	−	−	−	−	−	−	−	−	−	+	+	−	+	+
Oleic acid	17.7	2225	2184	−1.9	−	−	−	−	−	−	−	−	−	−	+	+	−	+	−	−	+	−
Stearic acid	18	2243	2198	−2	−	−	−	−	−	−	−	−	−	−	+	+	−	+	+	+	+	+
*n*-eicosanoic acid	22.5	2453	2388	−2.6	−	−	−	−	−	−	−	−	−	−	−	+	−	−	+	−	−	−
**TERPENES AND ISOPRENE DERIVATIVES**																						
Phytol	16.6	2041	2171	1.3	+	+	+	+	−	+	+	−	−	+	−	−	+	+	+	+	−	−
α-tocopherol	37.9	3094	3161	0.7	+	+	−	−	+	+	−	−	−	−	−	+	−	−	+	−	−	+
Stigmasterol	40.6	3229	3319	0.9	−	−	+	−	−	−	−	+	−	+	+	−	−	+	−	−	−	−
β-sitosterol	41.8	3289	3286	1	+	+	−	−	−	+	+	+	−	+	+	−	−	−	−	−	−	−
Lanosterol	43.2	3360	3391	0.3	+	+	−	−	+	+	+	+	+	−	−	−	−	−	−	−	−	−
Oleanolic acid	46.1	3500	3620	1.2	+	+	−	−	−	+	+	+	+	+	−	−	−	−	−	−	−	−
Ursolic acid	47	3546	3649	1	+	+	−	−	−	+	+	+	−	+	−	−	−	−	−	−	−	−
**FLAVONOIDS**																						
Catechin	32.5	2828	2864	0.4	+	+	+	+	+	−	+	+	−	+	−	+	−	−	+	−	+	−
Epicatechin	32.5	2828	2864	0.4	+	+	+	+	+	−	−	+	−	+	−	+	−	−	+	−	−	−
Epigallocatechin	33.5	2879	2915	0.4	+	+	−	−	−	−	−	−	−	−	−	−	−	−	−	−	−	−
Luteolin	36.5	3025	3078	0.5	−	−	+	+	−	−	−	−	−	−	−	−	−	−	−	−	−	−
Kaempferol	36.5	3025	3078	0.5	−	−	+	−	+	+	−	+	−	+	+	−	−	−	+	−	+	+
Quercetin	38.8	3141	3169	0.3	−	−	+	+	−	−	−	−	−	+	−	−	−	−	−	−	−	−
**OTHERS**																						
Guanosine	30	2706	2762	0.6	−	−	−	−	−	−	−	+	−	−	−	−	−	−	−	−	−	−
Dihydrocapsaicin	27.4	^*^	^*^		−	−	−	−	−	−	−	−	−	−	−	−	−	−	−	−	+	−
1-octacosanol	38.1	3110	3089	−0.3	−	−	−	−	−	−	−	−	−	−	−	−	+	+	+	+	+	+
1-triacontanol	42	3301	3315	0.2	−	−	−	−	−	−	−	−	−	−	−	−	+	−	+	+	+	−
Gluconic acid lactone 2	9.9	1716	1776	0.7	−	−	−	−	−	−	−	−	−	−	−	−	−	−	−	−	+	−

***1** C. grandiflora* leaves; ***2** C. grandiflora* stems; ***3*** leaves of *H. hebeclada* from P.E. Serra do Mar; ***4*** leaves of *H. hebeclada* from E.E. da Juréia; ***5*** stems of *H. hebeclada* from E.E. da Juréia; ***6** L. hoehnei* leaves; ***7** L. kunthiana* leaves; ***8** L. humilis* leaves; ***9** L. humilis* stems; ***10** P. excelsa* leaves; ***11** J. multifida* leaves; ***12** J. gossypifolia* leaves; ***13** S. swartizuanum* leaves from Cardoso; ***14** S. swartzianum* stems from Cardoso; ***15** S. swartzianum* leaves from Cunha; ***16** S. americanum* leaves; ***17** S. americanum* stems; ***18** S. excelsum* leaves.

### RAMSY deconvolution in GC-MS regions with low AMDIS-based *MF*

**Figure 2:**
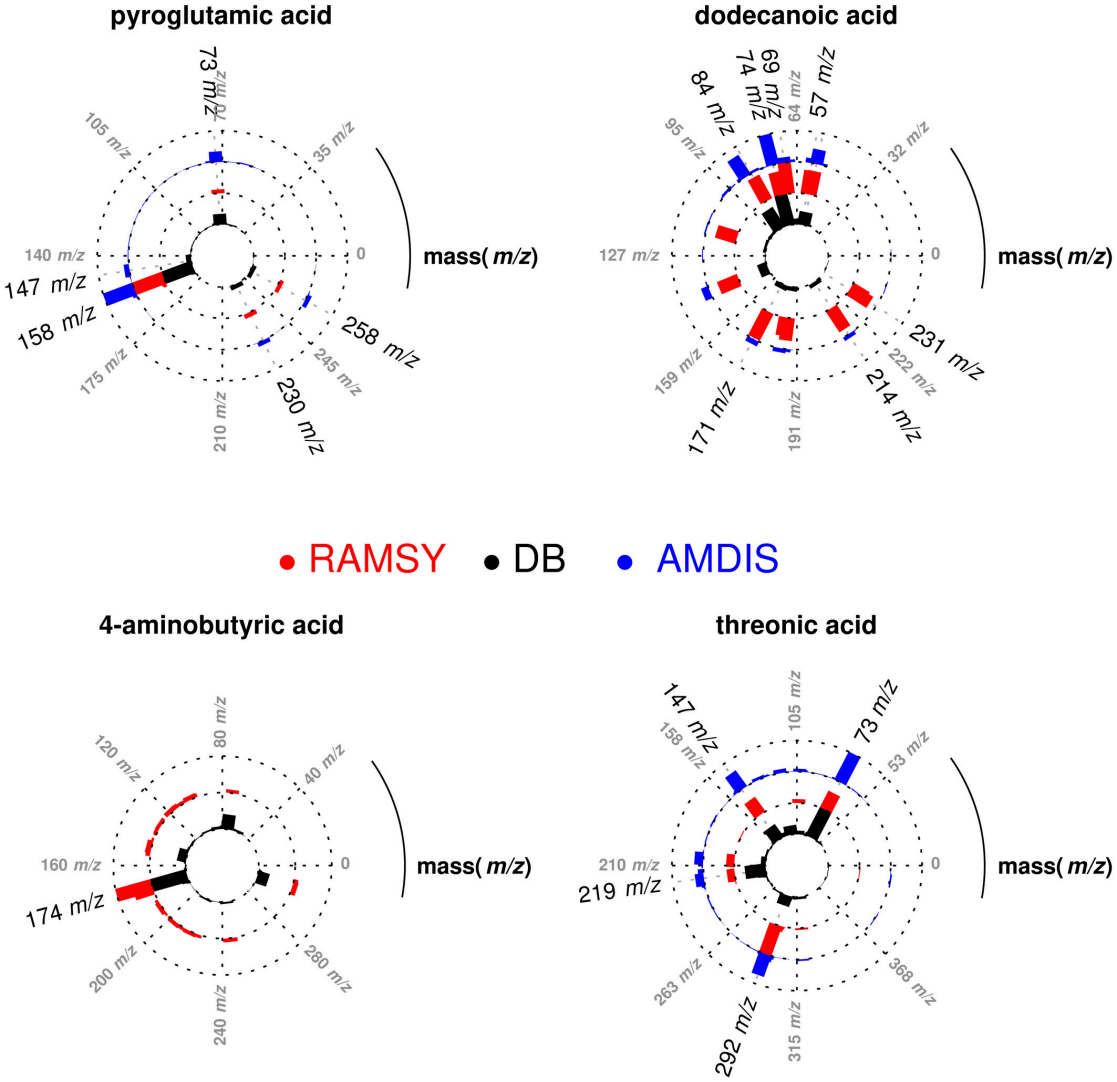
**Polar plot of “pure” MS spectra recovered from co-eluted GC-MS peaks between 7.45 and 7.60 min**. Each lane corresponds to the mass spectrum obtained from AMDIS (blue), RAMSY (red) or database-DB (black). Pyroglutamic acid (AMDIS *MF* = 750 and RAMSY *MF* = 970), dodecanoic acid (AMDIS *MF* = 810 and RAMSY *MF* = 920) and threonic acid (AMDIS *MF* = 710 and RAMSY *MF* = 920) were found using AMDIS and RAMSY, while 4-aminobutyric acid was found only by RAMSY (*MF* = 840).

After performing the optimization of AMDIS-based GC-MS dereplication, we applied RAMSY in regions that showed low *MF*s due to high peak overlap, low intensities and/or noise effects. In the first application of RAMSY, we selected a broad peak between 7.45 and 7.60 min for the *Couepia grandiflora* plant species sample (Chrysobalanceae). According to AMDIS, this peak represented the overlap of pyroglutamic acid (Rt 7.48 min, *MF* = 750), dodecanoic acid methyl ester from the FAME standard mixture (Rt 7.49 min, *MF* = 810) and threonic acid (Rt 7.53 min, *MF* = 710). We also observed the presence of unidentified low-intense ions 304 *m/z* and 174 *m/z*. We selected the 15 scans that formed the GC peak at 7.45–7.60 min for RAMSY analysis. The RAMSY spectrum calculation was performed using the fragment ion 156 *m/z* as the driving peak for pyroglutamic acid, 87 *m/z* for dodecanoic acid methyl ester, 292 *m/z* for threonic acid and 304 *m/z* for the unidentified compound. The averaged EI-MS spectrum was filtered with the RAMSY values (only those MS peaks with top RAMSY values were shown) and compared with MS libraries considering *MF*. RAMSY correctly identified ions from the three metabolites identified by AMDIS, as depicted in Figure 2. The application of RAMSY using 304 *m/z* as the driving peak provided a new mass spectrum suggested as 4-aminobutyric acid, based on the NIST MS database (*MF* = 840). These results provided evidence for the capabilities of RAMSY to recover low-intense ions in co-elution. RAMSY also provided *MF* values > 900 for the other compounds detected by AMDIS—dodecanoic acid methyl ester (*MF* = 920), pyroglutamic acid (*MF* = 970) and threonic acid (*MF* = 920), attesting to the ability of RAMSY to provide deconvolution enhancement.

**Figure 3:**
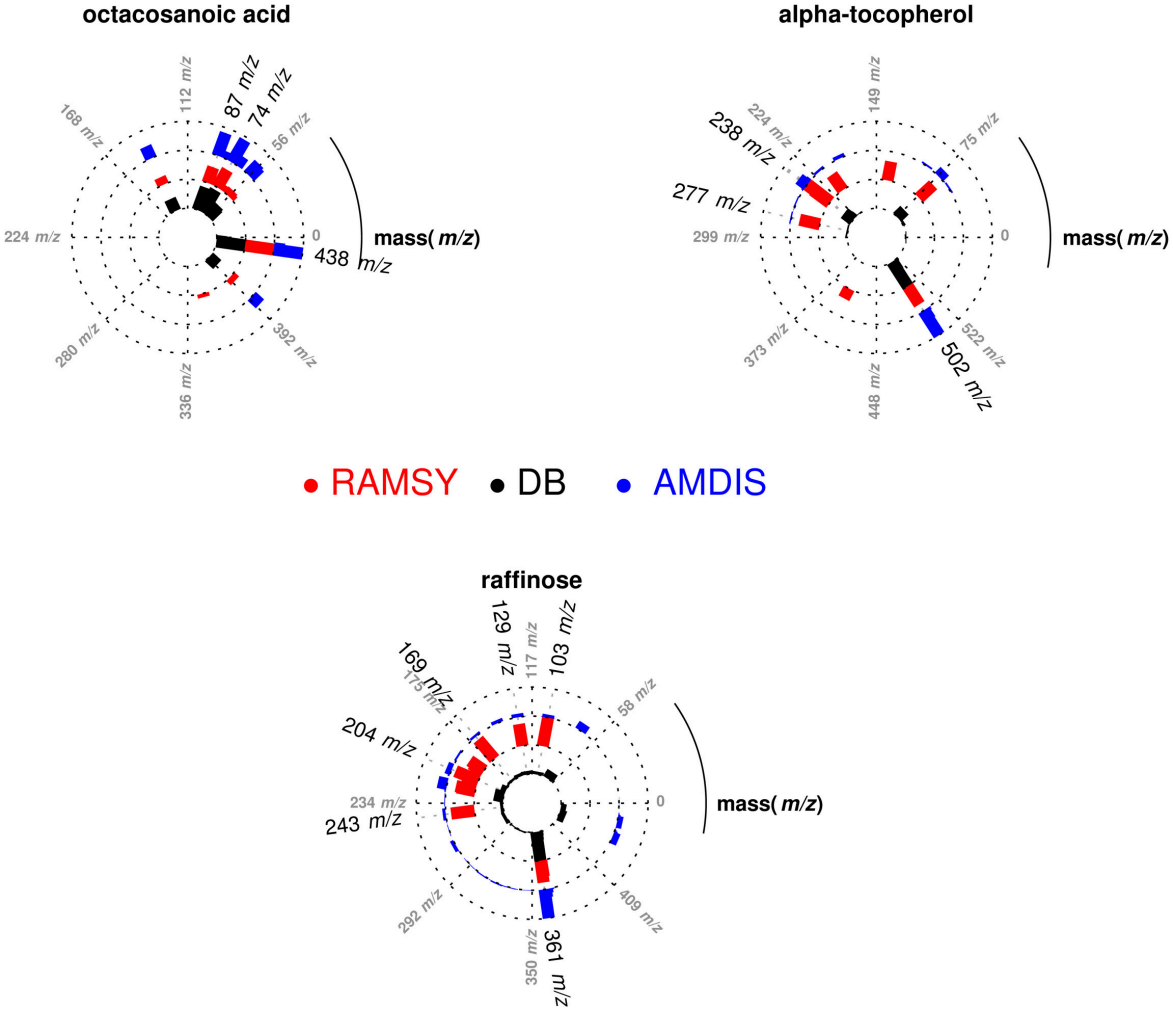
**Polar plot of “pure” MS spectra recovered from the co-eluted GC-MS peaks between 37.75 and 38.00 min**. Each lane corresponds to the mass spectra obtained from AMDIS (blue), RAMSY (red) or database-DB (black). Octacosanoic acid (AMDIS *MF* = 770 and RAMSY *MF* = 870), α-tocopherol (AMDIS *MF* = 500 and RAMSY *MF* = 900) and raffinose (AMDIS *MF* = 600 and RAMSY *MF* = 900) were found using AMDIS and RAMSY.

In another application of RAMSY, the chromatographic peak between 37.75 and 38.00 min observed in *Solanum americanum* led to the dereplication of three overlapped compounds by AMDIS, Figure 3. The molecules were identified as α-tocopherol (*MF* = 500), octacosanoic acid methyl ester (*MF* = 770) and raffinose (*MF* = 600). The RAMSY spectrum was calculated using the 26 scans that compose the GC-MS peak, by selecting driving peaks at 237 *m/z*, 87 *m/z* and 361 *m/z*. RAMSY improved the deconvolution, resulting in increased *MF* values for each of the metabolites: α-tocopherol (*MF* = 900), octacosanoic acid (*MF* = 870) and raffinose (*MF* = 900).

**Figure 4:**
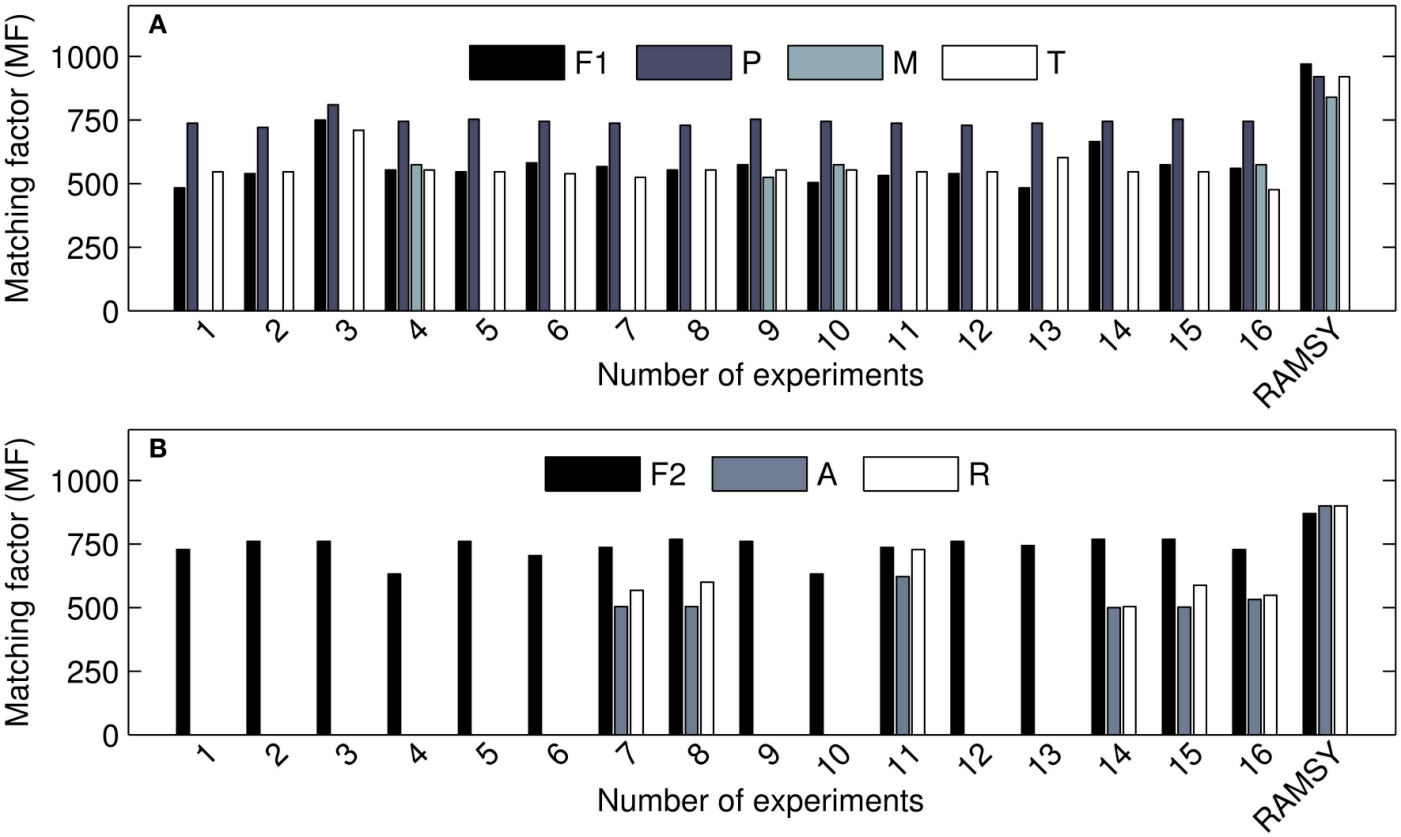
***MF* values for detected metabolites using AMDIS in different parameter sets (1–16) and RAMSY**. **(A)** represents the coeluted peak at 7.4–7.6 min; F1: C_12_ FAME std (dodecanoic acid); P: pyroglutamic acid; M: 4-aminobutyric acid; T: threonic acid. **(B)** represents the coeluted peak at 37.7–38.0 min: F2: C_28_ FAME std.; A: α-tocopherol; R: raffinose.

The comparison between RAMSY and AMDIS using the 2*v*^5−1^ optimization design experiments is shown in Figure 4 and indicates that the use of AMDIS alone consistently generated lower *MF* values and more false negatives. The application of RAMSY in combination with AMDIS assisted by CDF improved the identification of known metabolites in very complex biological samples.

**Figure 5:**
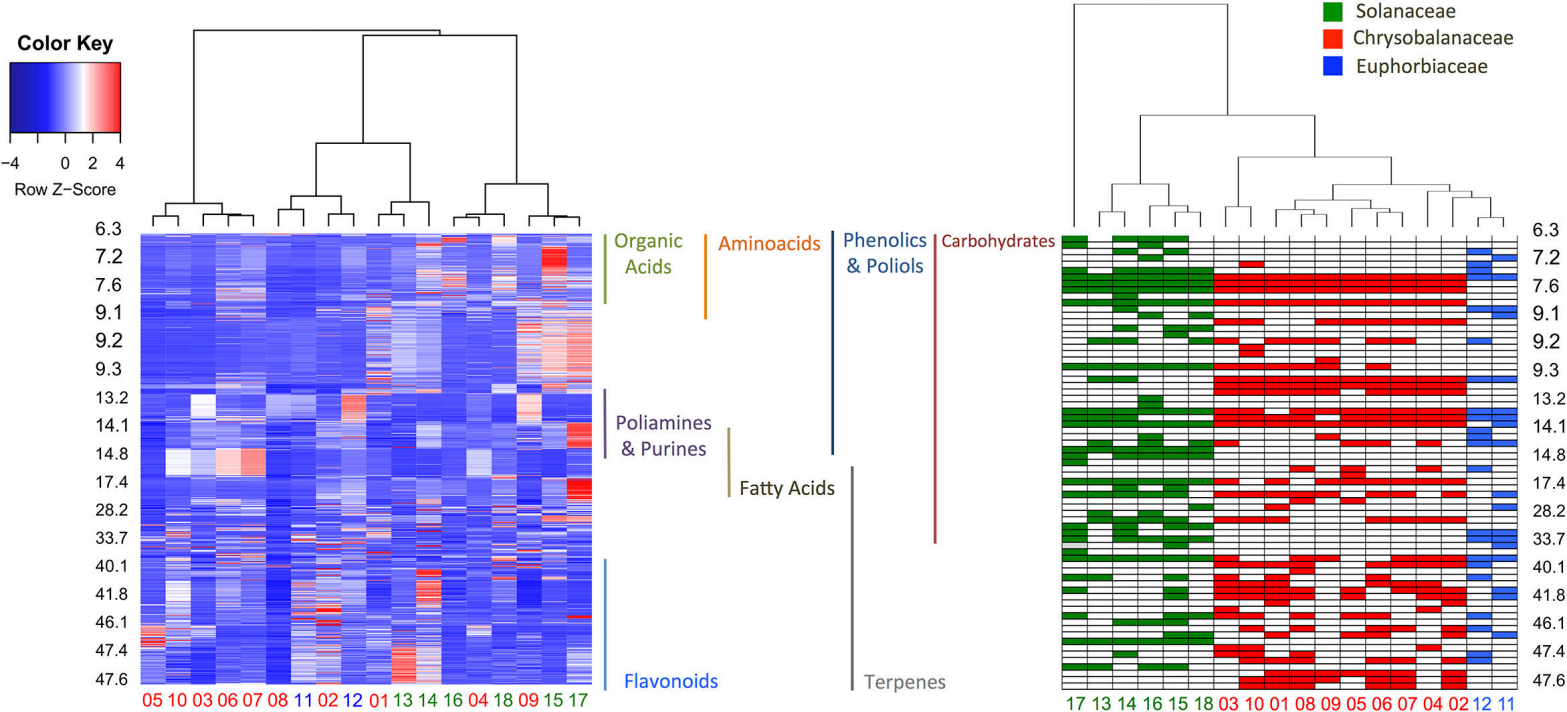
**HCA heatmap comparing the GC-MS raw data (left) and AMDIS-RAMSY-based identified metabolites (right)**. The GC-MS raw data heatmap (color key box—upper left) represents the metabolite concentrations, while the AMDIS-RAMSY color key indicates the presence among taxa families. The identified classes of metabolites are shown according to their retention times between the plots.

In order to evaluate the identification efficiency, we compared the GC-MS raw data and processed metabolite profiles (AMDIS-RAMSY) by HCA, as depicted in Figure 5. It is possible to observe a taxonomic correspondence based on the metabolic content from the GC-MS raw data and the dataset with the identified metabolites after AMDIS-RAMSY (colored spots). The AMDIS-RAMSY results presented better taxonomical grouping compared to raw data due to the spectral metabolic deconvolution and suppression of noise and interferent effects. According to the AMDIS-RAMSY taxonomical grouping, amino acids and fatty acids were responsible for distinguishing between Solanaceae species, while for the Chrysobalanaceae species the differentiation was based mainly on carbohydrates and flavonoids, as evidenced in Figure 5.

## Conclusions

Dereplication of natural products from GC-MS data was performed by combining two different deconvolution methods, AMDIS and RAMSY. According to the results, the optimization of AMDIS parameters using CDF can improve metabolite identification and reduce the number of false components. However, the empirical AMDIS method was not able to fully deconvolute all GC-peaks, leading to low *MF* values and/or missing metabolites, which justifies the application of a complementary method such as RAMSY. In this first use of RAMSY as a “digital filter” for AMDIS, it was possible to show improved ability of ratio analysis to recover low-intensity ions in co-eluted regions as well as the improvement of the deconvolution process and metabolite identification. The incorporation and automation of RAMSY jointly with AMDIS would benefit compound identification in mass spectra of complex biological mixtures, such as plants extracts. Additionally, the development of robust derivatized secondary metabolite libraries would assist in the identification of known metabolites, thereby improving the power of the dereplication tools shown here.

## Author contributions

FC and AP designed the work, performed the experiments, and wrote the manuscript. DS and RF contributed with data analysis and statistical methods, HG and DR assisted the experiments and performed the RAMSY approach, NL and IC wrote the manuscript. All authors listed, have made substantial, direct and intellectual contribution to the work, and approved it for publication.

### Conflict of interest statement

DR is an officer at Matrix-Bio, Inc. The other authors declare that the research was conducted in the absence of any commercial or financial relationships that could be construed as a potential conflict of interest.

## Abbreviations

AMDIS: Automated Mass Spectral Deconvolution and Identification System
RAMSY: Ratio Analysis of Mass SpectrometrY
LC-UV: Liquid Chromatography-UltraViolet detection
LC-MS: Liquid Chromatography Mass Spectroscopy
LC-MS/MS: Liquid Chromatography Tandem Mass Spectroscopy
GC-MS: Gas Chromatography Mass Spectroscopy
NAPRALERT: Natural Products Alert
CDF: Compound Detection Factor
HCA: Hierarchical Cluster Analysis.
